# Modification of the swirling well cell culture model to alter shear stress metrics

**DOI:** 10.1002/bit.28331

**Published:** 2023-01-20

**Authors:** Mehwish Arshad, Shuyu Cheng, Maarten van Reeuwijk, Spencer J. Sherwin, Peter D. Weinberg

**Affiliations:** ^1^ Department of Bioengineering Imperial College London London UK; ^2^ Department of Aeronautics Imperial College London London UK; ^3^ Department of Civil and Environmental Engineering Imperial College London London UK

**Keywords:** atherosclerosis, cross flow index, endothelium, hemodynamics, oscillatory shear index, transverse wall shear stress, wall shear stress

## Abstract

Effects of hemodynamic shear stress on endothelial cells have been extensively investigated using the “swirling well” method, in which cells are cultured in dishes or multiwell plates placed on an orbital shaker. A wave rotates around the well, producing complex patterns of shear. The method allows chronic exposure to flow with high throughput at low cost but has two disadvantages: a number of shear stress characteristics change in a broadly similar way from the center to the edge of the well, and cells at one location in the well may release mediators into the medium that affect the behavior of cells at other locations, exposed to different shears. These properties make it challenging to correlate cell properties with shear. The present study investigated simple alterations to ameliorate these issues. Flows were obtained by numerical simulation. Increasing the volume of fluid in the well‐altered dimensional but not dimensionless shear metrics. Adding a central cylinder to the base of the well‐forced fluid to flow in a square toroidal channel and reduced multidirectionality. Conversely, suspending a cylinder above the base of the well made the flow highly multidirectional. Increasing viscosity in the latter model increased the magnitude of dimensional but not dimensionless metrics. Finally, tilting the well changed the patterns of different wall shear stress metrics in different ways. Collectively, these methods allow similar flows over most of the cells cultured and/or allow the separation of different shear metrics. A combination of the methods overcomes the limitations of the baseline model.

## Graphical abstract

Cross‐sectional view of 1 well of a 12‐well plate, modified by the addition of a central polydimethylsiloxane cylinder, and the computational mesh used to analyze flow that is induced when the well is placed on an orbital shaker.

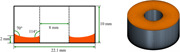

## 1 INTRODUCTION

Effects of hemodynamic wall shear stress (WSS) on arterial endothelial cells have received substantial attention because they are thought to account for the patchy distribution of atherosclerosis within the arterial system. Several shear stress characteristics have been implicated including not only high or low time average shear but also oscillatory shear, multidirectional shear, and shear with high spatial or temporal gradients (reviewed in Peiffer et al., 2013b; Weinberg, 2022). In vitro systems that expose endothelial cells to complex flows, as opposed to simple unidirectional or uniaxial flow, are consequently required.

The “swirling well” or “orbital shaker” method has been extensively used for this purpose (e.g., Arshad et al., 2021; Chakraborty et al., 2012; Dardik et al., 2005; Ghim et al., 2017; Potter et al., 2011; Warboys et al., 2014). Cells are cultured on the base of circular dishes or cylindrical wells in multiwell plates that are then placed on a horizontal platform which translates with a circular orbit in the plane of the platform. This motion forces a wave to rotate around the well, and spatially and temporally complex patterns of shear are produced at the base of the well. Corresponding cell behavior can be obtained using methods, especially those based on microscopy, that provide spatially resolved readouts. The patterns vary with well and orbital radii, orbital rotation rate, and depth and viscosity of the medium. Analytical and experimental methods for characterizing the flow are, respectively, approximate and difficult to implement so, starting with the work of Berson (Berson et al., 2008), the flow has generally been characterized by numerical methods; postprocessing gives maps of shear metrics over the base of the well. These can then be compared with maps of the behavior of the cells (reviewed in Warboys et al., 2019).

The system has several advantages: it produces complex flows, uses off‐the‐shelf components and allows chronic exposure with high throughput at low cost. The low volumes of fluid permit the use of expensive reagents and the analysis of conditioned medium, yet the number of cells is sufficient for “omics”‐type approaches (Ghim et al., 2018; Warboys et al., 2019). However, there are two significant disadvantages. One issue is that a number of shear stress characteristics change in step from the center to the edge of the well. For example, in the basic configuration used here, time average shear stress is constant from the center of the well to a radial distance of 7 mm center, where it starts rising, whereas how multidirectional the shear is during an orbit remains constant from the center until 7 mm and then falls (Arshad et al., 2022). Cells have more atheroprotective properties toward the edge, but at present, it is not possible to say whether high‐time average stress or low multidirectionality is responsible. A second issue is that cells at one location in the well, exposed to one type of flow, may release mediators into the medium that affect the behavior of the cells at other locations, experiencing other flows (Ghim et al., 2018, 2021); this corrupts the true correlation between applied shear and cell properties unless cells are grown only in one region (Ghim et al., 2018, 2021; Pang et al., 2021). Of course, this property can also be useful in identifying such mediators, which may be significant in vivo (Ghim et al., 2017, 2018, 2021).

The present study investigated the use of simple alterations to the swirling well model to overcome these disadvantages whilst maintaining the benefits. One aim was to change the way in which some shear properties vary across the well whilst leaving others unaltered. A second aim was to make the shear properties more uniform across the well whilst obtaining different properties in different wells. Three geometrical modifications were used: adding cylinders at the center of the well, either joined to the base or suspended above it, and tilting the well on the shaker platform. Additionally, changes were made to medium volume and viscosity—volume was changed in the basic (Control) model and the Tilted model, and viscosity was changed in the Suspended Cylinder (SC) model.

These methods were chosen for the following reasons. An increase of medium volume should stretch the vertical velocity profile and thus reduce shear at the base, whilst increasing viscosity should, conversely, increase the level of shear stress corresponding to each shear rate. (Viscosity additionally has the effect of damping wave breaking, but this was expected to have a smaller influence.) Attaching a cylinder to the base prevents radial flow over the central portion of the well. Hence, shear in the outer portion should become more tangential and less radial. Conversely, the SC confines radial flow to a narrow portion of the vertical plane, and this should increase the extent of radial flows over the base. Finally, tilting the well was incorporated to break radial symmetry. The expectation was that symmetry would be broken in different ways for different shear metrics, thus allowing separation of their effects on cells.

The results are discussed with respect to preliminary tests of their practicality (Arshad, 2021) and earlier studies that have altered the model (Driessen et al., 2020; dela Paz et al., 2012).

## 2 METHODS

### 2.1 Flow simulations

Simulations were run in STAR‐CCM+ (v11.02.010‐r8) using the Volume of Fluid model. Results for the Control model have previously been validated by particle image velocimetry (Arshad et al., 2022). The liquid was assigned a density of 1003 kg/m^3^; a dynamic viscosity of 0.78 × 10^−3^ Pa.s, appropriate for 37°C, was used unless stated otherwise. The remaining geometrical space was filled with an air of density 1.1115 kg/m^3^ and dynamic viscosity 18.688 × 10^−6^ Pa.s. The base and sides of the well and the surfaces of added cylinders were defined as no‐slip boundary conditions. The open surface was assigned a constant (atmospheric) pressure. The simulations were run in the frame of reference of the orbital shaker platform, making the well stationary.

An acceleration of [*aω* ^2^cos(*ωt*), −*aω* ^2^sin(*ωt*), −9.81] was applied in the *x*, *y*, and *z* planes, with orbital radius *a* = 5 mm, and angular velocity *ω* = 15.7 rad/s, as used in many experiments (Alpresa et al., 2018a; Arshad et al., 2021, 2022; Ghim et al., 2017, 2021; Potter et al., 2011). The *z* term is the acceleration due to gravity. The free surface was defined at volume fraction of water equal to 0.5. To incorporate surface tension, the Multiphase Interaction Model was activated. An Interface Momentum Dissipation value of 3.0 was used. (See Arshad et al. [2022] for a justification of this choice.).

The simulation was initiated from a static state. A time step of 5 × 10^−5^ s was selected to satisfy the CFL < 0.5 criterion, except in the tilted well models where it was 1 × 10^−4^ s. Each time step was iterated until asymptotic convergence of the solution, defined as a <1 × 10^−5^ Pa change over five iterations, was achieved. Simulations were run for a minimum of 8 cycles and the reported data were obtained from the last cycle. Further details are given in Supporting Information.

Dimensionless groups used in this study were Eccentricity (E), Forcing (F), Shallowness (Γ), Reynolds number (Re), Bond number (Bo), and Weber number (We) (Alpresa et al., 2018a; Arshad et al., 2022) with the following parameters: orbital forcing radius *a*, angular velocity of the orbital shaker *ω*, well radius *R*, gravitational acceleration *g*, average fluid height *d*, density of water $\rho_{w}$, density of air $\rho_{a}$, surface tension σ, linear velocity *U*, and kinematic viscosity of the fluid ν:

(1) $$\begin{array}{l} E=\frac{a}{R},F=\frac{a\omega }{g},\Gamma =\frac{d}{R},\text{Re}\;=\frac{R^{2}\omega }{\nu },\mathrm{Bo}=\frac{\left(\rho_{w}-\rho_{a}\right)g{\left(2R\right)}^{2}}{\sigma },\\ \mathrm{and}\;\mathrm{We}=\frac{\rho_{w}U^{2}(2R)}{\sigma }. \end{array}$$

The choice of the first four dimensionless groups is explained in our earlier work (Alpresa et al., 2018a), which also describes their influence on the fundamental behavior of the model (particularly resonance, wave breaking and uncovering of the bottom of the well). Bo and We indicate, respectively, the importance of gravitational acceleration and inertia compared to surface tension. *R*, the radius of the well, was used as a characteristic length when calculating Bo and We for the Control model; for the models with a central cylinder (CC), it was replaced by the channel width (*R*_well_ − *R*_cylinder_). The linear velocity at the edge of the well was used for U in all cases and hence the maximum value of We over the well radius was obtained. Parameters that differed between models and dimensionless groups are given in Table 1.

**Table 1:** Parameters and dimensionless numbers of the CFD models analyzed in this study

Models	Parameters			Dimensionless groups				
	*ν* (m^2^/s) × 10^−6^	*d* (mm)	*R* (mm)	Bo	We	Re	E	Γ
Volume								
Control	0.78	2	11.05	67	9	2466	0.45	0.18
1151 µl	0.78	3	11.05	67	9	2466	0.45	0.27
1534 µl	0.78	4	11.05	67	9	2466	0.45	0.27
Geometry								
SC 1 mm	0.78	5	2.05	4	2	85	2.44	2.44
SC 2 mm	0.78	5	2.05	4	2	85	2.44	2.44
CC	0.78	2	7.05	42	6	1004	0.71	0.28
Geometry + viscosity								
SC + 2.2 mm^2^/s	2.2	5	2.05	4	2	30	2.44	2.44
SC + 3.4 mm^2^/s	3.4	5	2.05	4	2	19	2.44	2.44
SC + 4.0 mm^2^/s	4.0	5	2.05	4	2	17	2.44	2.44
Tilt								
767 µ + 2.5^◦^	4.0	2	11.05	67	9	2466	0.45	0.18
767 µ + 5^◦^	4.0	2	11.05	67	9	2466	0.45	0.18
1534 µ + 5^◦^	4.0	4	11.05	67	9	2466	0.45	0.36
1534 µ + 10^◦^	4.0	4	11.05	67	9	2466	0.45	0.36

*Note*: The omitted F = 0.13, RPM = 150, *ω* = 15.7 Rad/s, *U* = 0.174 m/s, *a* = 5 mm, *ρ_wr_* = 1003 kg/m^3^, *ρ_a_* = 1.1115 kg/m^3^, *g* = 9.81 m^2^/s, and *σ* = 0.07 mN/m were kept constant.

Surface tension and wetting were incorporated into the models despite Bo and We being >1 because our previous study (Arshad et al., 2022) showed that wave‐breaking behavior becomes unrealistic if they are entirely omitted. Note that surface tension was assigned different values for different models. All models with added cylinders used a value of 47 mN/m, which is appropriate for culture medium with added serum at 37°C, as used in most experiments with endothelial cells (Arshad et al., 2022). (The proteins in the serum have surfactant properties.) The narrow gaps between the walls of the cylinder and well may make accurate values of surface tension important. In the Control model, and the Control model with increased volumes of liquid, 72 mN/m was used. This value is appropriate for water or unsupplemented cell culture medium at 37°C. The Control model has been used for measurements of endothelial permeability (e.g., Ghim et al., 2017); serum, which causes cell division, is omitted or much reduced in such experiments. We have previously shown that changing the value of surface tension does not alter fundamental flow behavior in the Control model (Arshad et al., 2022), so the results will also be applicable to experiments where serum is added to the medium. Finally, it was not practicable to include surface tension or wetting in the tilted models. This issue is discussed below.

Geometries were as follows:

#### 2.1.1 Control model

The geometry, representing 1 well of a 12‐well plate, consisted of a cylindrical well with 22.1 mm diameter, 10 mm height, and an open surface. The well was filled with liquid to an average depth of 2 mm. The geometry was discretized into 612,500 structured 3D hexahedral cells. The geometry and mesh are shown in Supporting Information: Figure 1a. A contact angle of 70° was used; it is appropriate for polystyrene, which is used to make culture plasticware, and was obtained from our earlier measurements (Arshad et al., 2022).

#### 2.1.2 Model with increased volume of liquid

The volume of liquid was increased from 767 µl in the control model to 1151 or 1534 µl, corresponding to average depths of 3 and 4 mm.

#### 2.1.3 Models with altered geometry

The geometry was changed by the addition of cylindrical objects. Contact angles of 70° and 114° were used at the well wall and cylinder wall, respectively. The value of 114° applies to polydimethylsiloxane (PDMS) (Pitts et al., 2013), which is moldable and sterilizable and, therefore, widely used in cell culture experiments. PDMS was used to construct the cylinders in our tests of these models, described below.

##### CC

A solid vertical cylinder of diameter 8 mm was placed centrally in the well, attached to its base. The volume of liquid was adjusted to maintain an average depth of 2 mm. The height of the cylinder was greater than the maximum elevation of the free surface above the base of the well. The geometry and mesh with 316,800 cells are shown in Supporting Information: Figure 1b.

##### SC

A solid vertical cylinder of diameter 18 mm was placed centrally in the well, suspended 1 or 2 mm above its base. The bottom edge of the cylinder was rounded (radius of curvature 0.2 mm). The average liquid depth was adjusted to 5 mm to prevent uncovering of the base of the cylinder when the fluid was in motion. The geometries, and meshes with 505,600 and 608,000 cells, respectively, are shown in Supporting Information: Figure 1c,d.

#### 2.1.4 SC models with increased viscosity

The model with the SC and 2‐mm gap was modified by increasing the viscosity of the liquid from its control value of 0.78 × 10^−3^ to 2.2 × 10^−3^, 3.4 × 10^−3^ or 4.0 × 10^−3^ Pa.s. Since density is only slightly altered in experiments using dextran as a viscosity‐increasing agent, as discussed in (Arshad et al., 2022), density was kept at its control value in the simulations.

#### 2.1.5 Tilted models

The base of the Control model was tilted by 2.5° or 5° and a mesh with 679,680 or 573,440 cells, respectively, was used. Additionally, the volume of liquid in the control model was increased to 1534 µl and the base of the well was tilted to 5° or 10°; meshes with 573,440 and 600,000 cells, respectively, were used. The increased volume was required to prevent uncovering of the base at the highest tilt angle.

### 2.2 Postprocessing of flow simulations

Shear stress acting on the base of the well is here termed “wall shear stress” (WSS) to be consistent with the terminology used for intact blood vessels. The instantaneous WSS components at each mesh point on the base of the well were extracted at every 10th‐time step. In the basic model and most of the modified versions, every point that is at the same distance from the center of the well experiences the same temporal pattern of instantaneous WSS magnitude and direction, although there is a phase shift from one circumferential location to the next. For these models, WSS metrics were postprocessed using a MATLAB (2016a) script. Symmetry in the azimuthal direction is abolished in the tilted well. The entire WSS distribution at the base of the well has to be mapped out over one cycle. This was performed using a VTK Python script and visualized using Paraview (v.5.6.0).

Seven established metrics were calculated. The first five were: time average wall shear stress (TAWSS), which is the mean over the cycle of the magnitudes of the instantaneous WSS vectors; the magnitude of the mean of the instantaneous WSS vectors (MagMeanWSS); the oscillatory shear index (OSI) (He & Ku, 1996), which uses a ratio of the first two metrics to determine how much the instantaneous WSS vectors deviate from the direction of the mean WSS vector (including instantaneous vectors that are in the opposite direction to the mean vector, along the same axis); transverse WSS (transWSS) (Peiffer et al., 2013a), which is the time average of the components of the instantaneous WSS vectors that are perpendicular to the mean WSS vector, and the cross‐flow index (CFI) (Mohamied et al., 2017), which is related to transWSS but takes into account only the angle of the instantaneous vectors and not their magnitude.

(2) $$\mathrm{TAWSS}=\frac{1}{T}\int_{0}^{T}|{\overset{⃗}{\tau }}_{\omega }|\mathrm{dt},\text{where}\;|{\overset{⃗}{\tau }}_{\omega }|\equiv \sqrt{\tau_{x}^{2}+\tau_{y}^{2}+\tau_{z}^{2}},$$

(3) $$\mathrm{MagMeanWSS}=|{\overset{⃗}{\tau }}_{\mathrm{mean}}|\text{where}\;{\overset{⃗}{\tau }}_{\mathrm{mean}}=\frac{1}{T}\int_{0}^{T}{\overset{⃗}{\tau }}_{w}\mathrm{dt},$$

(4) $$\mathrm{OSI}=\frac{1}{2}\left(1-\frac{\left|\int_{0}^{T}{\vec{\tau }}_{\omega }\mathrm{dt}\right|}{\int_{0}^{T}\left|{\vec{\tau }}_{\omega }\right|\mathrm{dt}}\right)=\frac{1}{2}\left(1-\frac{\mathrm{MagMeanWSS}}{\mathrm{TAWSS}}\right),$$

(5) $$\mathrm{TransWSS}=\frac{1}{T}\int_{0}^{T}\left|{\vec{\tau }}_{\omega }.\left(\vec{n}\times \frac{{\vec{\tau }}_{\mathrm{mean}}}{\left|{\vec{\tau }}_{\mathrm{mean}}\right|}\right)\right|\mathrm{dt},$$

(6) $$\mathrm{CFI}=\frac{1}{T}\int_{0}^{T}\left|\frac{{\vec{\tau }}_{\omega }}{\left|{\vec{\tau }}_{\omega }\right|}.\left(\vec{n}\times \frac{{\vec{\tau }}_{\mathrm{mean}}}{\left|{\vec{\tau }}_{\mathrm{mean}}\right|}\right)\right|\mathrm{dt},$$

where $\tau_{x}$, $\tau_{y}$, and $\tau_{z}$ are the *x*, *y*, and *z* components of the instantaneous WSS vector, ${\vec{\tau }}_{w}$, *t* is the time increment, *T* is the time it takes to complete one cycle, and $\vec{n}$ is the unit vector perpendicular to the base of the well.

Two additional metrics, the minimized transverse WSS (transWSSmin) (Ghim et al., 2017) and the minimized CFI (CFImin) (Mohamied et al., 2017), generalize the transWSS and CFI, respectively, by computing the transWSS and CFI using as a reference orientation not the direction of the mean WSS vector but the direction that minimizes the transWSS or CFI:

(7) $$\mathrm{TransWSSmin}\leftarrow \min_{\emptyset \in \left[0,2\pi \right]}\frac{1}{T}\int_{0}^{T}\left|{\vec{\tau }}_{\omega }.\left(\vec{n}\times \left(\begin{array}{l} \cos\emptyset \\ \sin\emptyset \\ 0 \end{array}\right)\right)\right|\mathrm{dt},$$

(8) $$\mathrm{CFImin}\leftarrow \min_{\emptyset \in \left[0,2\pi \right]}\frac{1}{T}\int_{0}^{T}\left|\frac{{\vec{\tau }}_{\omega }}{\left|{\vec{\tau }}_{\omega }\right|}.\left(\vec{n}\times \left(\begin{array}{l} \cos\emptyset \\ \sin\emptyset \\ 0 \end{array}\right)\right)\right|\mathrm{dt},$$

where ∅ is the angle that is altered so as to minimize the computed metric. A conventional assumption is that endothelial cells align with the mean WSS vector. TransWSS and the CFI then represent, respectively, the cycle average WSS acting across rather than along the cell, and the magnitude‐independent equivalent of the same metric. However, we have shown that endothelial cells can align so as to minimize the flow across them (Arshad et al., 2021). TransWSSmin calculates the cycle average WSS acting across cells showing such behavior and, by analogy, the CFImin is the magnitude‐independent equivalent.

Finally, four directions were computed: the direction of the mean WSS vector; the direction of the modal WSS vector; and the directions that minimize transWSS and the CFI (Equations 7 and 8). To obtain the modal WSS, the instantaneous WSS vectors occurring at each point over one cycle were divided into 18 bins according to their orientation, and the most commonly occurring angle over one cycle was computed.

## 3 RESULTS

### 3.1 Data presentation

WSS data for nontilted wells were plotted in a number of different ways to show different features of the flow. The types of plots are explained first.

**Figure 1:**
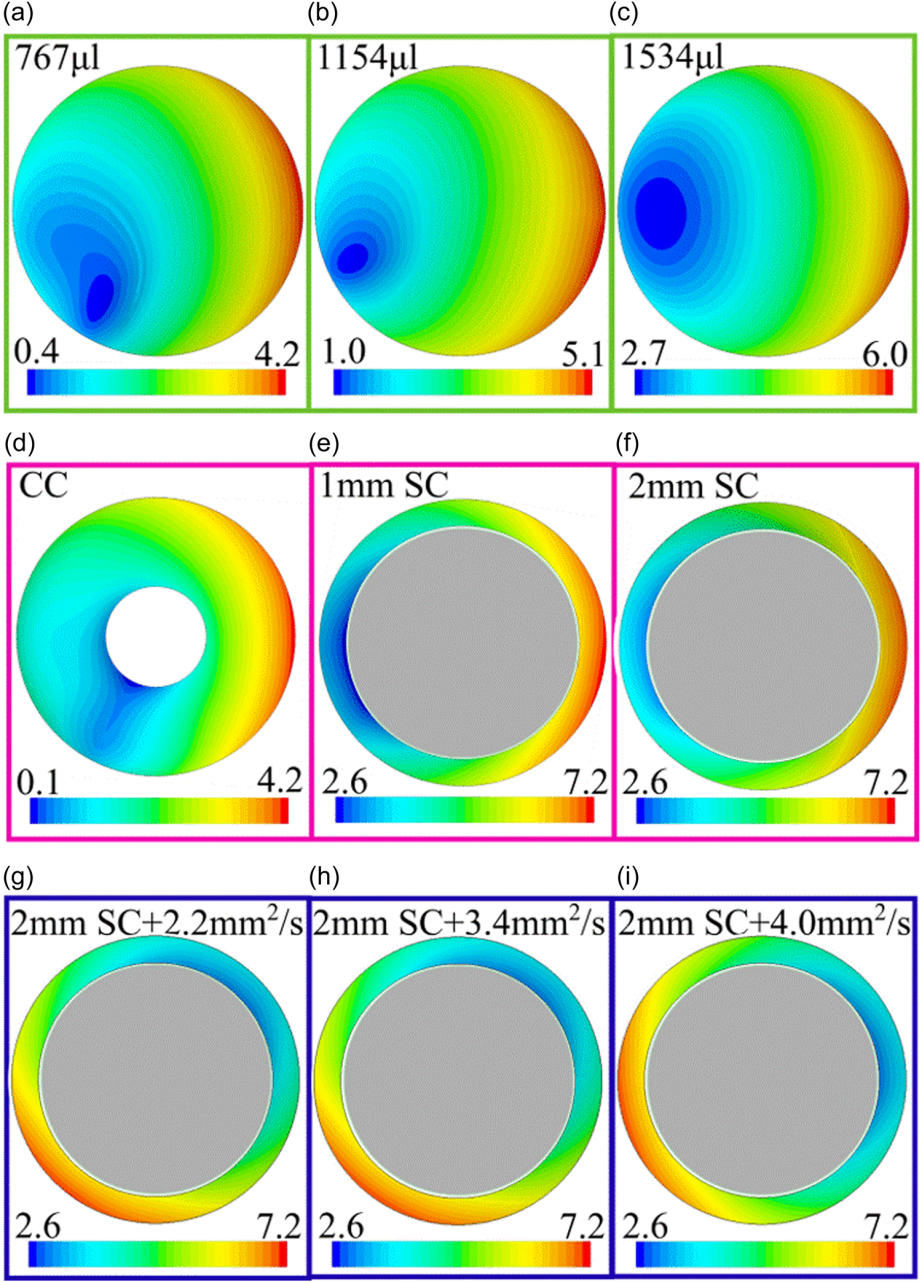
Free surface elevation maps for the nontilted models. (Heights on color bars are in mm.) (a)–(c) Control model and models with increased volume. (d)–(f) Models with a central cylinder (CC) or a suspended cylinder (SC) with a 1 or 2 mm gap between the cylinder base and the well base. (g)–(i) Suspended cylinder model with 2 mm gap and increased viscosities.

The height of the free surface above the base of the well is mapped in Figure 1.

**Figure 2:**
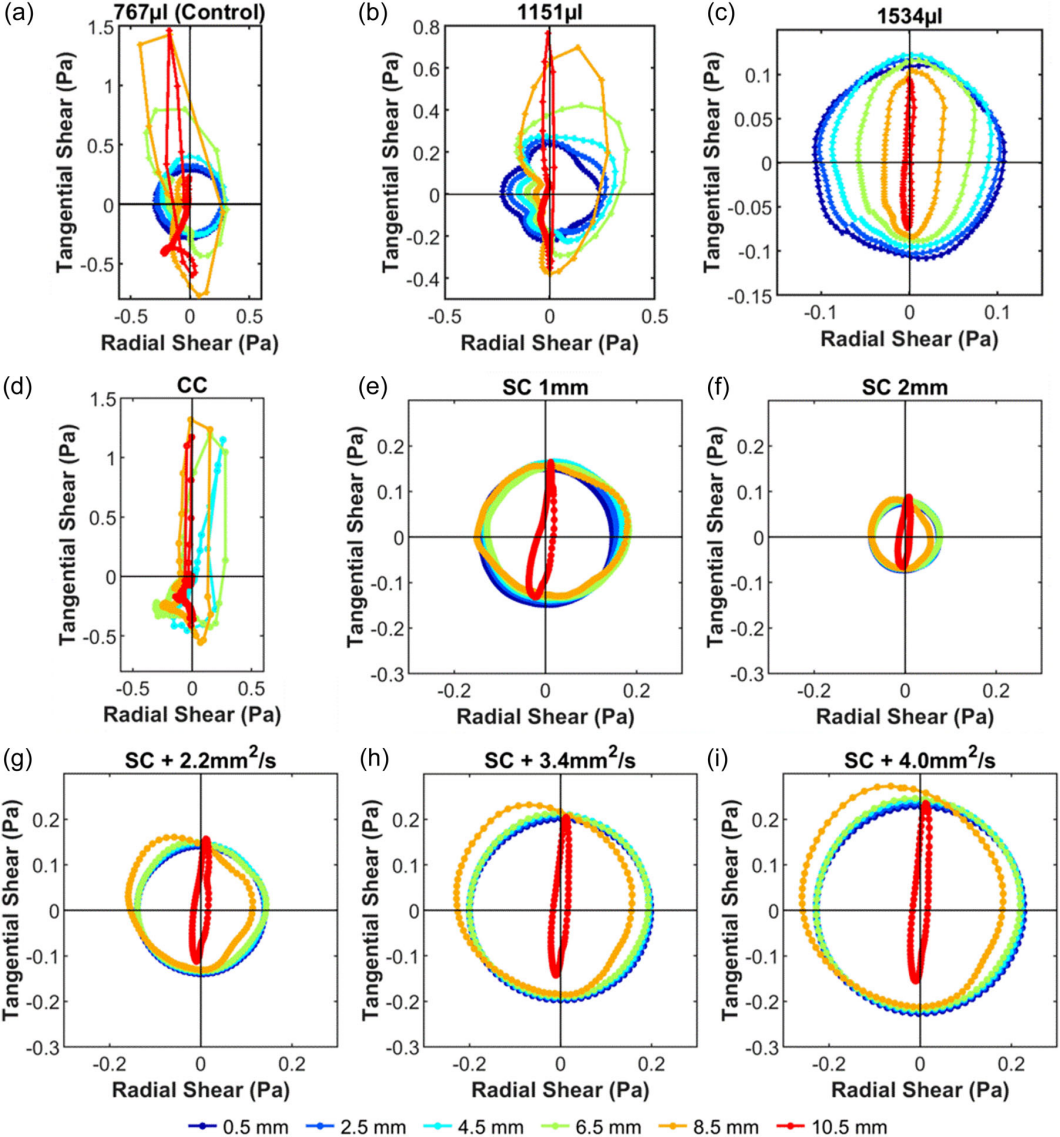
Polar plots of the instantaneous WSS magnitude and direction as a function of radial distance from the center of the well. The lines show the locus of points representing the tip of the instantaneous WSS vector, which has its origin at 0,0. (Note that 0,0 does not designate the same real position for each differently colored line, but the point for which the vectors were calculated.) The filled circles on each plot are spaced at intervals of 5 ms. Negative radial values indicate vector components pointing in the direction of the well center, and negative tangential values indicate components in the direction of the swirling wave. (a) Control model, (b) volume increased to 1151 µl, (c) volume increased to 1534 µl, (d) Central Cylinder (CC) model, (e) Suspended Cylinder (SC) model with 1 mm gap, (f) Suspended Cylinder model with 2 mm gap, (g) Suspended Cylinder model with 2 mm gap, and viscosity increased to 2.2 mm^2^/s, (h) 3.4 mm^2^/s, or (i) 4.0 mm^2^/s. Note the different magnitude scales.

Polar plots (Figure 2) show the direction and magnitude of the instantaneous WSS vectors throughout one cycle for selected locations along a radius on the base of the well. Each line is the locus of points representing the location of the tip of the vector over time; the vector has its origin at 0,0 on the graph. (Note that this is not necessarily the center of the well.) On the radial axis, negative values indicate locations towards the center of the well; on the tangential axis, negative values indicate the dominant direction of wave travel. Dots along the line show equal time intervals.

**Figure 3:**
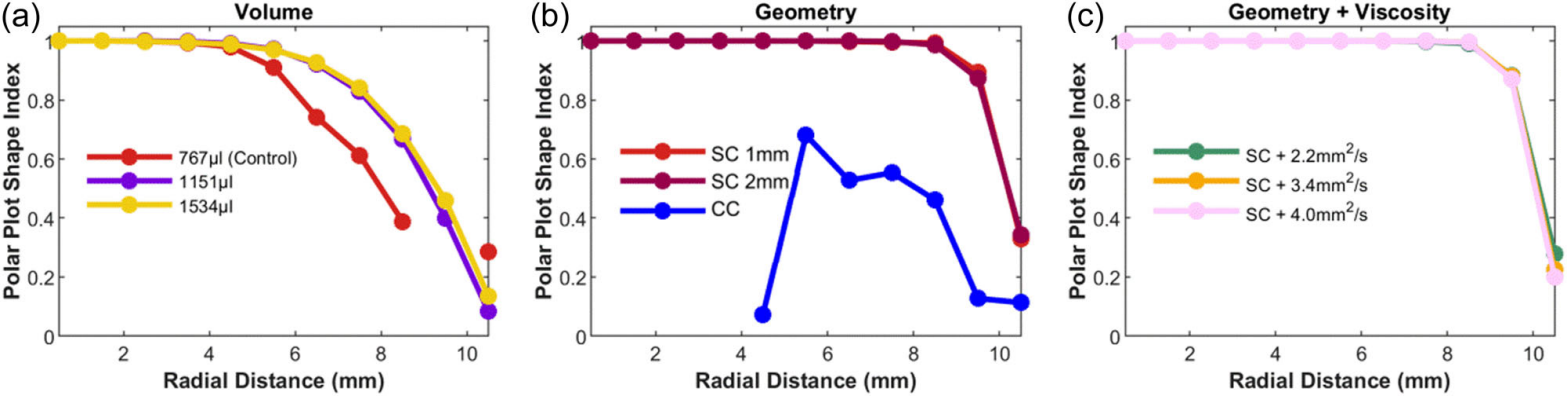
An ellipse was fitted to polar plots like those in Figure 2 and the Shape Index (SI) for each ellipse was plotted as a function of radial distance from the center of the well. SI of a circle = 1. SI of a straight line = 0. (a) Control model and models with increased volume. (b) Models with central cylinder, or suspended cylinder with 1 or 2 mm gap between the base of the cylinder and the base of the well. (c) Models having a suspended cylinder with 2 mm gap and increased viscosity. (The algorithm would not fit an ellipse to the highly elongated polar plot for the Control model at 9.5 mm.)

Figure 3 quantifies the degree of circularity (i.e., multidirectionality) of polar plots such as those in Figure 2 by fitting an ellipse and then computing its shape index (SI) (Cornhill et al., 1980):

(9) $$\text{SI}\;=4\pi A/P^{2},$$

where *A* = area and *P* = perimeter. SI has values of 1 for a circle and 0 for a straight line.

**Figure 4:**
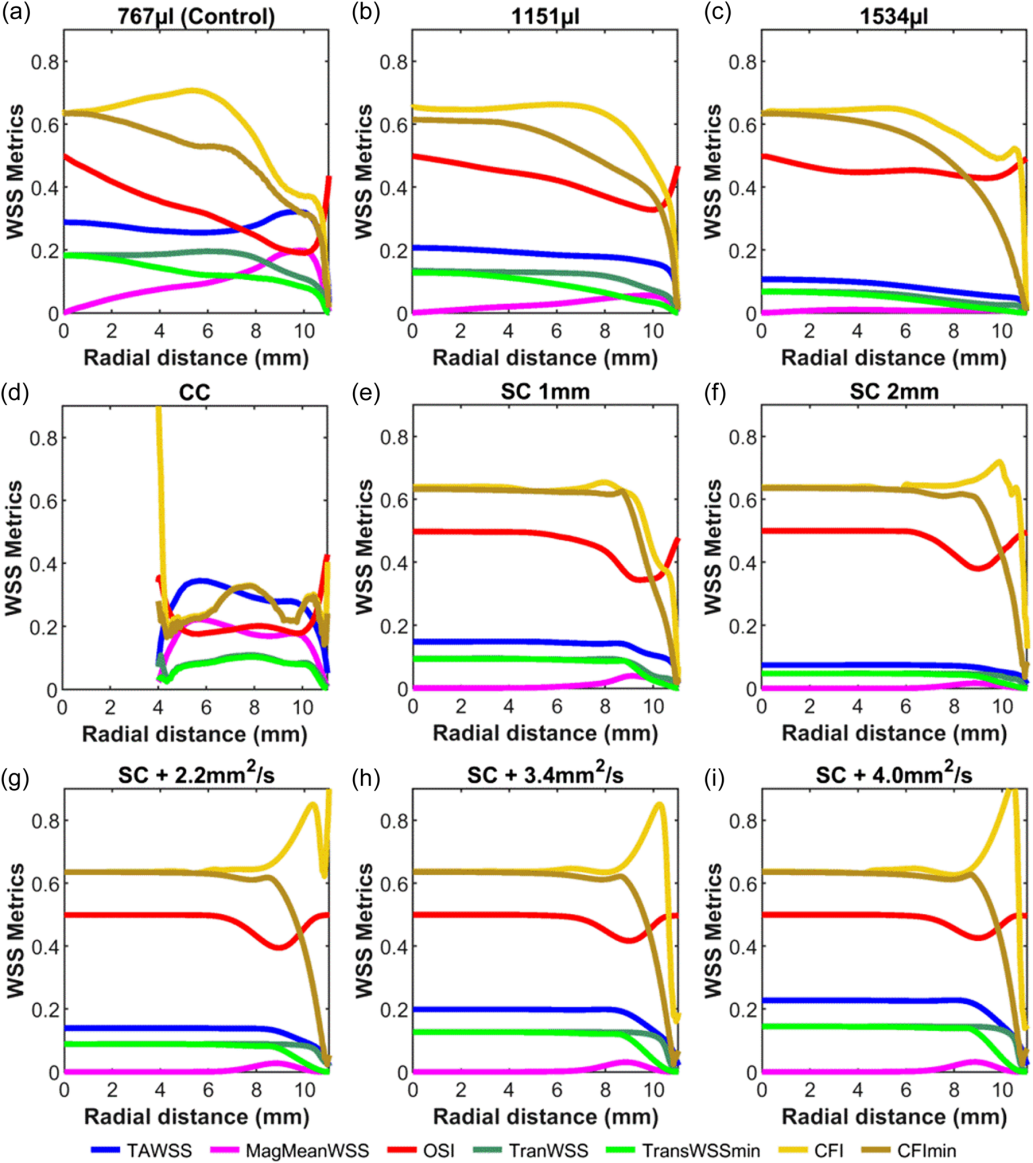
WSS metrics as a function of radial distance from the center of the well for the different models: (a) Control model and (b,c) Control model with increased volume; (d) Central Cylinder model and (e,f) Suspended Cylinder model with 1 or 2 mm gap; (g,h,i) Suspended Cylinder model with increased viscosity. Values for dimensional metrics are in Pascal. WSS, wall shear stress. CC, central cylinder; SC, suspended cylinder

Radial plots (Figure 4) show the values of the seven WSS metrics defined above as a function of distance from the center to the edge of the well.

The mean and modal WSS direction and the orientations that minimize transWSSmin and CFImin are plotted as a function of radial distance in Supporting Information: Figure 4 and are only briefly discussed below.

**Figure 5:**
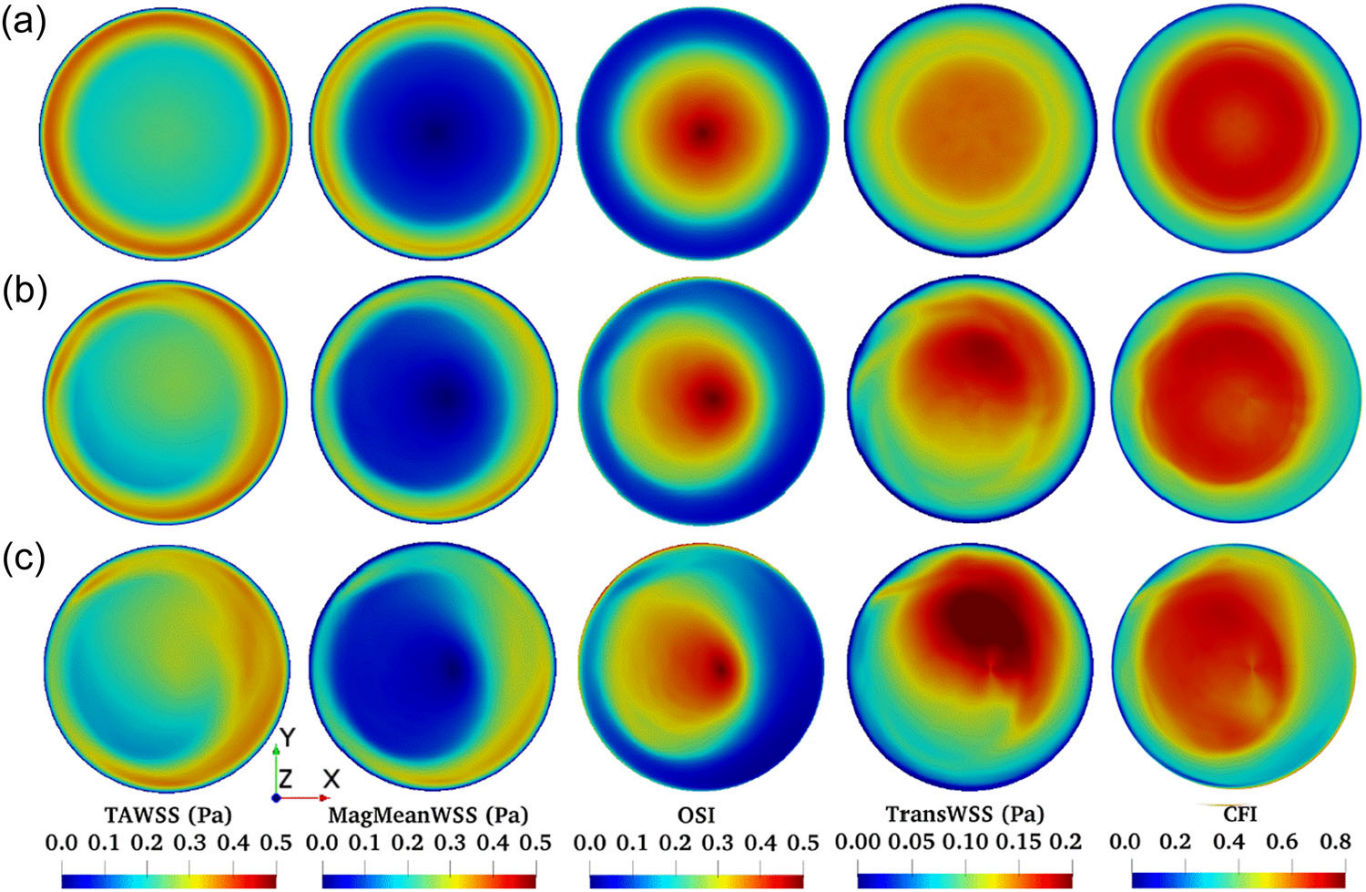
WSS metrics mapped over the base of the 767 µl model with an incline of (a) 0° (equivalent to the Control model) or (b) 2.5° or (c) 5°. The highest point of the base is at the right of the maps. WSS, wall shear stress.

**Figure 6:**
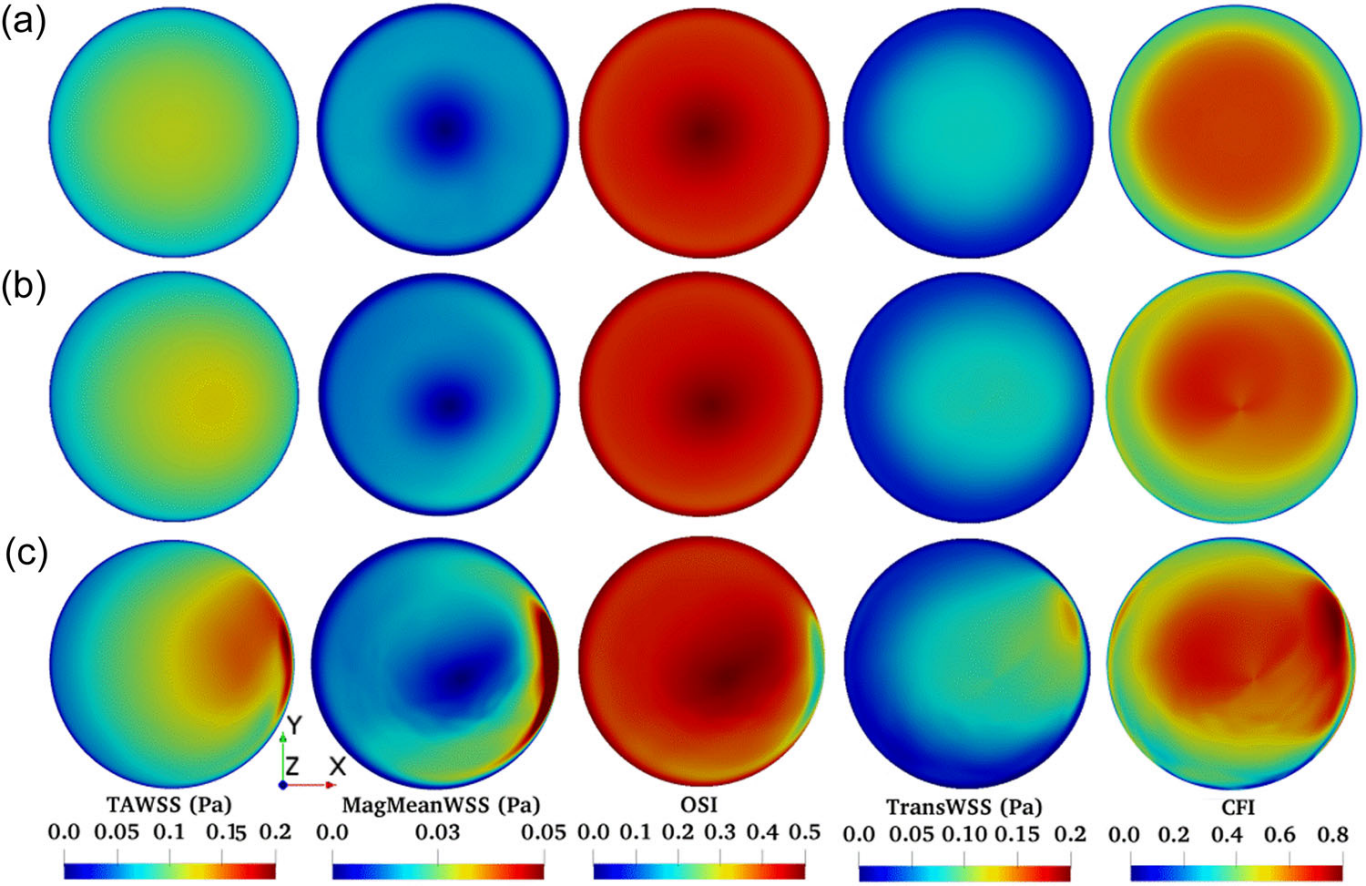
WSS metrics mapped over the base of the 1534 µl model with an incline of (a) 0°, (b) 5°, or (c) 10°. The highest point of the base is at the right of the maps. WSS, wall shear stress.

**Figure 7:**
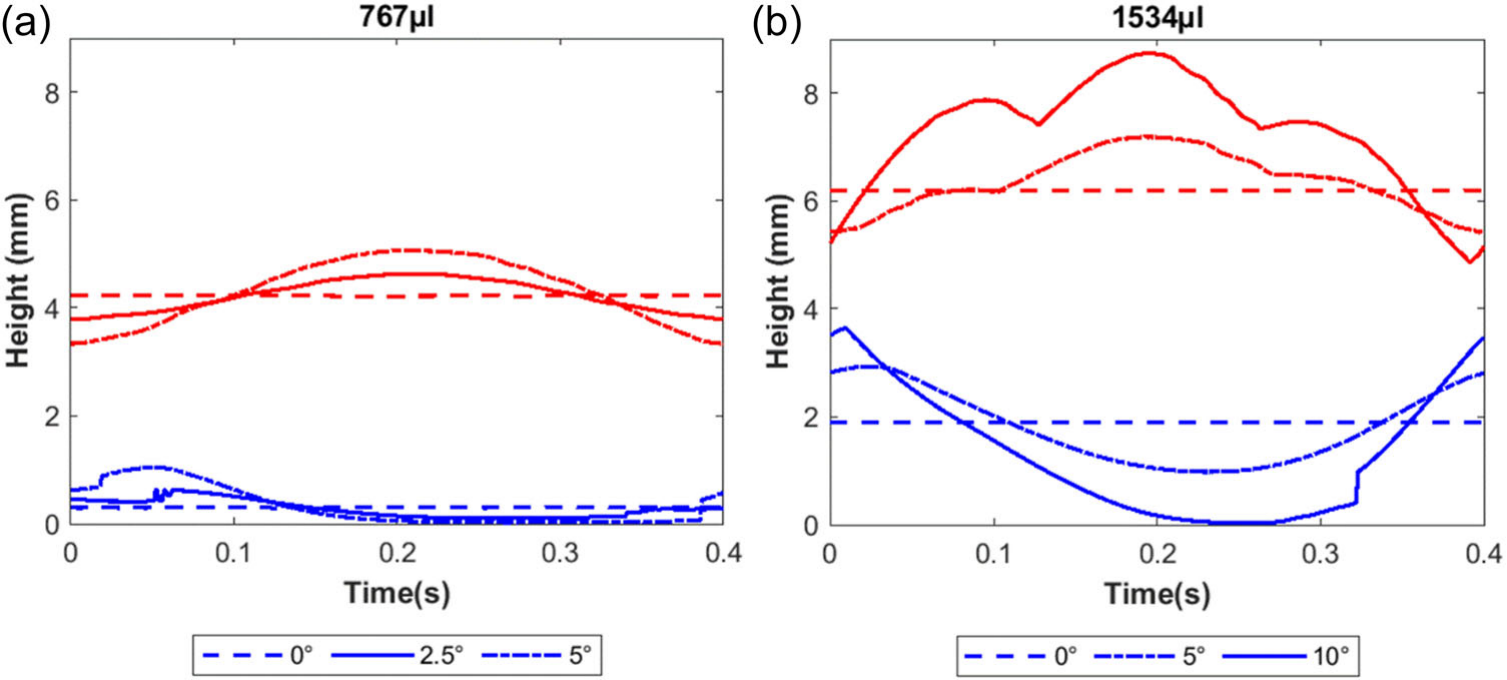
Minimum (blue lines) and maximum (red lines) fluid elevation within the well over one cycle of the shaker for the models described in Figures 5 and 6.

The plots described above assume radial symmetry, albeit with shifts in phase, and are therefore inapplicable to flow in the tilted well, for which 2‐D maps of shear metrics are given in Figures 5 and 6 and height information is presented in Figure 7.

### 3.2 Control model and models with increased volume of liquid

The map of liquid height in the Control model (Figure 1a) demonstrates that the free surface is inclined from one side of the well to the other; it does not have a central minimum as would be produced by a vortexer, for example. The minimum and maximum heights were both larger in models with increased volume (Figure 1b,c; note the different color bar scales) but the models remained in the shallow water regime (Alpresa et al., 2018a)—fluid height normalized by well radius was always <1 (Table 1)—and the overall patterns were similar to the baseline model, the only visible change being a slight skewing of contours around the lowest heights.

The polar plot for the Control model (Figure 2a) shows the pattern identified in previous studies, wherein the flow is more multidirectional at the center than the edge. The dark blue line is the plot for a radial location close to the center of the Control model. Since the flow in this case is purely multidirectional—that is, the instantaneous vector sweeps through 360° at a regular rate and with constant magnitude—the plot is a circle centered on the origin and the dots are equally spaced.

The red line shows the behavior for a location close to the edge of the well. (The origin of the vector is still assigned a location of 0,0 although in physical space it is displaced from the center of the well.) In this case, the flow is nearly bidirectional: forwards and backwards along an axis close to the tangential direction, spending more time in the forward than backward direction. Correspondingly, the plot shows a narrow shape with greater tangential than radial magnitudes, and there are more negative than positive points.

When the volume of fluid in the well was increased from 767 to 1151 µl and then 1534 µl, increasing average height from 2 to 3 mm and then 4 mm, the plots remained approximately circular near the center of the well and highly elongated near the edge (indeed this trend became clearer with increasing height—see Figure 3a) but tangential and radial magnitudes decreased, and those near the edge decreased more than those near the center, perhaps due to increased wave dampening, so that the magnitude of the vectors at the edge never exceed those near the center (Figure 2b,c; note the changes of scale).

The plots of WSS metrics as a function of radial location (Figure 4a–c) confirm the substantial drop in TAWSS as volume was increased and further show that the peak in TAWSS that occurred at radii >8 mm in the Control model was abolished. For the 1534 µl model, TAWSS was virtually constant across most of the well. Similar trends were seen in MagMeanWSS.

Considering the dimensionless metrics related to the multidirectionality of flow, the OSI was at its theoretical maximum of 0.5 in the center of the Control model and was lower at greater radial distances, except close to the edge. CFI and CFImin broadly fell with increasing radial distance, like the OSI, consistent with a switch from multidirectional to uniaxial flow. When the volume was increased, however, the curves for all three indices became flatter, remaining high for a greater proportion of the well. Indeed, OSI was effectively constant across the entire well. This behavior is consistent with the shape of the polar plots, quantified in Figure 3a: as volume was increased, the plots remained more nearly circular for greater distances from the center.

TransWSS and transWSSmin in the Control model were high (approximately 2/3rds of TAWSS) at the center and the plots had the same shape as those for the CFI and CFImin curves, respectively. That remained true as volume increased. However, transWSS and transWSSmin scale with TAWSS. Because TAWSS dropped as volume increased, so did the magnitudes of these metrics.

### 3.3 Models with altered geometry

#### 3.3.1 CC

The surface height map for the model with a CC joined to the base of the well (Figure 1d) shows a similar pattern and similar absolute magnitudes to the control model in regions not occupied by the cylinder itself; slopes are visible in the radial as well as the tangential direction.

Although the height map does not indicate a uniform wave circulating around the square toroidal channel, the polar plot (Figure 2d) does suggest a tendency towards this type of flow. The orange and red lines (8.5 and 10.5 mm) are even more elongated than the corresponding lines in the Control model, and the turquoise and green lines (4.5 and 6.5 mm) now also show this type of behavior. This change is quantified by the Shape Indices (compare the red line in Figure 3a with the blue line in Figure 3b).

In the radial plot of WSS metrics (Figure 4d), the no‐slip condition imposed at the cylinder wall forces dimensional metrics to zero at *r* = 4 mm, whereas the dimensionless metrics stay constant (CFImin) or rise (CFI, OSI) at that location. In the rest of the channel cross‐section, TAWSS and MagMeanWSS peak near the cylinder wall (5 mm), and the remaining metrics, which characterize multidirectionality, peak further out. Magnitudes of TAWSS and MagMeanWSS are broadly comparable to values in the outer part of the Control model but OSI, transWSS, and transWSSmin are generally lower, and CFI and CFImin are much lower, collectively indicating that there is less change in the flow direction.

#### 3.3.2 SC

The SC had a larger radius than the CC. The 1 or 2 mm gap between the base of the cylinder and the base of the well was completely filled with liquid; there was no free surface in this region, and the surface height maps (Figure 1e,f), therefore, show only a narrow ring at the edge of the well. This makes it hard to compare them with the Control model. Nevertheless, as with the CC model, radial as well as tangential variations in free surface height are apparent.

A striking feature of the polar plots for the SC models (Figure 2e,f) is that for all radial locations under the cylinder (0–8.5 mm), they were nearly circular (Shape Index of 1—see Figure 3b) and centered on the origin, indicating purely multidirectional flow. Only the red line, representing a radial distance of 10.5 mm, which is not under the cylinder, was elongated. Magnitudes for the SC model with 2 mm gap were smaller than those for the Control model, and those for the SC model with 1 mm gap were smaller still.

Consistent with this pattern, WSS metrics were constant for at least the central 6–8 mm of the 9 mm radius of the cylinder (Figure 4e,f). OSI, CFI, and CFImin retained their high values, and MagMeanWSS its low value, found at the center of the Control model. Outside the cylinder, CFI and CFImin dropped rapidly, whereas OSI did not and MagMeanWSS rose, consistent with the flow in this region reversing but not having radial components.

TAWSS was reduced by nearly half with the 1 mm gap and by 2/3rds with the 2 mm gap. TransWSS and transWSSmin, which are scaled by TAWSS, were consequently also reduced. Outside of the cylinder, TAWSS fell, presumably as the height of the liquid increased, and transWSS and transWSSmin accordingly also fell.

### 3.4 SC models with increased viscosity

Maps of surface height at viscosities of 2.2, 3.4, and 4.0 mm^2^/s (Figure 1g–i) are indistinguishable from one another and from the SC model with baseline viscosity (Figure 1f).

The same is true for the shape of the corresponding polar plots (Figure 2g–i cf. Figure 2f) and Shape Indices (Figure 3c cf. Figure 3b). However, the magnitude of the instantaneous WSS vectors increased with each increase in viscosity, although there was a less than proportional change (Figure 3f–i).

Similarly, increasing viscosity had no effect on the shape of any of the radial plots of WSS metrics (Figure 4f–i), and no effect on the magnitude of the dimensionless OSI, CFI, and CFImin. Corresponding to the increased magnitude of the instantaneous WSS vectors, the magnitude of TAWSS increased, and accordingly, so did transWSS and transWSSmin. (MagMeanWSS also increased, although that is hard to discern on the graph.) Thus altering viscosity is a method for increasing values of dimensional WSS metrics whilst leaving values of dimensionless metrics essentially unaltered.

### 3.5 Tilted models

When the base was horizontal, WSS metrics were symmetrical around the center of the well, regardless of the volume of fluid (Figure 5a, equivalent to the Control model, and Figure 6a, with increased volume), but this symmetry was broken by tilting the base of the well at increasing angles (Figures 5b,c and 6b,c).

Maximum values of the TAWSS and MagMeanWSS occurred at, or in the vicinity of, the highest point of the base (corresponding to the right of the maps). Of the metrics indicating multidirectionality, the same trend was seen in dimensional transWSS, whereas minimum values of the dimensionless OSI were seen in or near this region. The dimensionless CFI showed more complex properties, behaving like OSI in the lower volume Tilted model and like transWSS in the higher volume one; complexity is expected because all flow behavior contributing to the CFI will also contribute to the OSI, whereas the reverse is not true (purely oscillatory flow generates OSI but not CFI), and because transWSS is affected by the level of TAWSS, but that is not true for the OSI or CFI. TAWSS magnitudes were lower at the higher volume (note the change in color bar scale), while OSI was substantially higher as well as more uniform.

The maximum and minimum height of the free surface above the base of the well was constant over the cycle for the horizontal cases, but fluctuated with tilt, the degree of fluctuation increasing with the angle of tilt (Figure 7).

## 4 DISCUSSION

The aim of this study was to increase the utility of the swirling well system by developing simple methods for increasing the separability of WSS metrics or reducing their variation across the well whilst maintaining the inherent simplicity, low cost and high throughput of the technique. The baseline model, used in several previous studies (Alpresa et al., 2018a; Arshad et al., 2021, 2022; Ghim et al., 2017, 2021; Potter et al., 2011), was a single well of a 12‐well plate with 2 mm average medium depth, rotated at 150 rpm in a circular orbit of 10 mm diameter. Effects of increasing viscosity in the Control model have been reported in a previous paper (Arshad et al., 2021). In that study, the magnitude of all dimensional WSS metrics—TAWSS, MagMeanWSS, transWSS and transWSSmin—increased with viscosity but plots of the metrics versus radial distance from the well center retained approximately the shape seen without enhanced viscosity. (The increase in magnitude was less pronounced close to the wall of the well, perhaps because there was some damping of the wave and/or an increase in boundary layer thickness.) The dimensionless metrics—OSI, CFI, and CFImin—did not show the general increase in magnitude, but their relative magnitudes were altered towards the edge, again consistent with damping or boundary layer effects. This method could be used to increase WSS generally, and to distinguish effects of dimensional versus dimensionless metrics on cells.

The modifications investigated in the present study were: increasing the volume of fluid in the well, adding a central cylinder (CC) to the base of the well or a suspended cylinder (SC) above it, increasing viscosity in the SC model, and tilting the well on the shaker. When the volume of fluid was increased, plots of WSS metrics versus radial distance from the center of the well broadly maintained their shape but absolute magnitudes were decreased, especially towards the edge of the well. As with altered viscosity, this method can therefore be used to separate the effects on cells of dimensional metrics (such as TAWSS and transWSS), which decreased, from the effects of dimensionless metrics (such as OSI and CFI), which stayed approximately constant. Note that the magnitudes of the dimensional metrics were increased by increasing viscosity but decreased by increasing volume, extending the range that can be examined.

Adding a CC to the base of the well forced fluid to flow in a square toroidal channel. (A toroid is a surface of revolution with a hole in the middle, a square toroid is one with a square cross‐section, and a square toroidal channel is a square toroid with an open surface.) The multidirectionality of the flow was reduced in this region compared to the same radial locations in the Control model: transWSS, the CFI, and the OSI all decreased even though TAWSS remained the same or increased. MagMeanWSS also increased, consistent with a reduction in the mutual canceling of instantaneous vector components in opposite directions. This method can therefore be used to examine the effects of a nearly uniaxial flow over most of the area where cells grow.

We are aware of two previous attempts to use this method. dela Paz et al. (2012) grew cells in a 100 mm‐diameter culture dish; a CC was created by gluing a 60 mm‐diameter dish inside it. However, only the maximum WSS was estimated, and it was approximated using the extended solution to Stokes second problem; this method is not accurate when applied to the swirling well system, which violates several of the underlying assumptions (Alpresa et al., 2018b).

Driessen et al. (2020). subjected this design to a systematic study, varying rotational rate, liquid height, and diameters of the orbit and the inner and outer dishes. WSS metrics were obtained by numerical methods similar to ours. However, there was little overlap in system parameters and WSS metrics with the present study. The nearest combination was an 89 mm dish used with a 56 mm‐diameter cylinder, 10 mm orbit, 200 rpm rotational speed, and 3 mm liquid depth. The only metric that can be compared is the OSI, although their definition of it excluded the initial constant in Equation (4) so their values have been halved here. They obtained a maximum OSI of approximately 0.05 halfway across the channel, decaying to zero at either boundary, whereas we obtained 0.2 in the middle, rising to 0.4 at either boundary. The most likely explanation for the discrepancy is the different geometry, because OSI at the edge changed from negligible to high in their simulations of dishes without a CC when dish diameter was increased from 35 to 134 mm, even when all other parameters remained the same.

Suspending a cylinder above the base of the well made the flow highly multidirectional everywhere under the cylinder. Over most of the diameter of the cylinder, the CFI and OSI retained magnitudes normally seen only at the center of the well. If the polar plot were perfectly circular, then the CFI and CFImin would be 2/π (approximately 0.64), and the transWSS and transWSSmin would be (2/π × TAWSS. These values were attained for the majority of the radius under the cylinder. The theoretical maximum value of the OSI is 0.5 and that was also attained. MagMeanWSS was low, as expected for purely multidirectional flow. This method, in contradistinction to the CC model, could be used to expose cells to highly multidirectional flow over the majority of the well.

Note that the CFI can be bigger than 2/π, and transWSS can be bigger than (2/π) × TAWSS, if there are large instantaneous WSS vectors in nearly opposite directions and the mean direction thus becomes a smaller vector approximately perpendicular to them. CFI values greater than 2/π are clearly discernible in the polar plot for the SC model at radii >8 mm, and can be seen even in the plot for the Control Model. The OSI, however, cannot exceed 0.5.

The SC model additionally reduced absolute WSS magnitudes under the cylinder: transWSS and TAWSS were low, more so with the 2‐mm gap than the 1‐mm gap. Since our previous study (Arshad et al., 2021) showed that increasing viscosity in the Control configuration raised all dimensional WSS metrics, viscosity was increased in the SC model to see whether magnitudes could be recovered. As expected, magnitudes were again increased. The effects were modest, but this method could be used to distinguish between effects on cells of dimensional and dimensionless indices of multidirectionality (transWSS vs. CFI and transWSSmin vs. CFImin).

Alterations produced by tilting the well had a different character. TransWSS and the CFI could be increased to levels not seen in the baseline configuration whilst the OSI was not markedly increased, allowing discrimination of the influences of these properties. However, the main effect was that the plots lost their radial symmetry, and different WSS metrics were shifted in different directions. The maximum OSI moved towards the left of the map (the lowest point) and the maximum transWSS to the top right, whilst the highest TAWSS and MagMeanWSS moved to the right or bottom right. This symmetry breaking should allow additional discrimination provided that spatially resolved measurements of cell properties can be made over the whole base of the well, and are not restricted to single transects across the well.

A limitation is that surface tension could not be incorporated in numerical simulations of the tilted well. In the Control model, omitting viscosity leads to the appearance of breaking waves that are not seen in the physical model (Arshad et al., 2022). In the tilted models, however, the fluid layer is extremely shallow in places and wave breaking would therefore be expected even in simulations that did incorporate viscosity. We speculate that including viscosity might alter the precise nature of the shifts described above but would not make them disappear.

Although the mean and modal vector directions and the orientations that minimized the CFI and transWSS were not the main focus of the present study, we draw attention to a few key findings. First, the orientations that minimized the CFI and transWSS at any radial location were similar to each other. Second, the CC, which reduced multidirectionality, made all four orientations similar to one another. Third, the SC, which increased multidirectionality, made the modal direction highly unstable; presumably, the number of instantaneous vectors occupying each “bin” was similar and only small fluctuations were necessary to change the bin that held the most. The Control Model and the models with increased volume showed steady trends and gave clear differences between the metrics.

We next consider the physiological relevance of the WSS values obtained in the various models. Our computations for the human right coronary artery (Kandangwa et al., 2022) give maximum values of approximately 0.2 for OSI, 0.4 Pa for transWSS, 0.5 for CFI, and 4.5 Pa for TAWSS. Hence the values obtained in Figures 5 and 6 are appropriate other than for TAWSS, where they are too low. However, this is highly dependent on vessel. Cheng (2003). used MRI to measure TAWSS in the human abdominal aorta and obtained values of 0.14–0.2 Pa at rest, which are similar to the levels achieved in the tilted well.

A number of practical considerations need to be taken into account when implementing these models for cell culture. The volume of medium cannot be increased beyond a value that slows the diffusion of oxygen and carbon dioxide to and from the cells sufficiently to harm them. The critical volume would need to be determined for each model, since swirling the well will increase mass transport. The increased volumes described above, in conjunction with our standard well size and orbital shaker settings, are compatible with successful cell culture (Arshad, 2021).

A similar issue applies with the SC, which reduces the area of the free surface and increases mass transport distances for cells under the cylinder itself. Although there is a continuous cross‐flow under the cylinder, there will be a maximum cylinder diameter and minimum gap height beyond which it would be necessary to use hollow cylinders with a porous base.

Materials for cylinders need to be biocompatible, as does any adhesive that comes into contact with the medium. We have successfully cultured cells (Arshad, 2021) using cylinders made from PDMS, which can be molded into any desired dimensions using 3D‐printed or machined molds. It is the material of choice in microfluidic applications (McDonald et al., 2000). The hydrophobic surface of PDMS allowed it to attach strongly to the polystyrene lid of the multiwell plate without glue. It also attached without glue to of the base of the well, even during prolonged submersion, when this was plasma‐treated glass rather than plastic.

We have increased the viscosity of the medium in earlier studies by adding dextran, a polysaccharide that has been used for this purpose by others (Blackman et al., 2002; Qiu & Tarbell, 2000; Tardy et al., 1997). There have been conflicting reports about whether this treatment affects cellular mechanosensitivity and viability (Arshad, 2021; Dancu et al., 2007; Rouleau et al., 2010). Compatibility therefore needs to be checked for each cell type and set of culture conditions.

Tilting the wells is eminently practicable—it can be achieved simply by putting a spacer between one edge of the culture dish and the shaker platform. However, the well may need an increased volume of medium to cover all the cells throughout the cycle. The maximum tilt of 5° at the 767 µl volume gave a minimum free surface height of only 26 µm (Figure 7a). When the volume within the well was increased to 1534 µl, a higher incline of 10° could be applied, although the minimum height was still only 38 µm (Figure 7b).

Our preliminary cell culture data (Arshad, 2021, fig. 6.11) illustrate the utility of the SC and CC models. The orientation of the nucleus of porcine aortic endothelial cells was measured as a function of radial distance within the well. After 7 days of static conditions, all nuclear orientations were equally represented at all radial locations in the SC model. That is, with no shear, the cells were randomly oriented. When shear was applied for the same duration, the same result was obtained. This contrasts with our data for the Control model (Arshad et al., 2021), in which shear caused alignment and orientation that varied substantially with radial location, and presumably results from the multidirectional nature of the flow produced by the SC; there is no dominant flow direction. With the CC model, there was again no preferred orientation under static conditions, but there was a clear preferred orientation under shear, consistent with the more uniaxial flow produced in the square toroidal channel. Hence the modifications to the well are sufficient to produce dramatically different endothelial behaviors.

Finally, we consider whether the modifications to the Control model described here are more broadly applicable. The study of Driessen et al. (2020), for example, shows substantial differences in shear patterns between dishes of different sizes in the baseline configuration. Nevertheless, it seems reasonable to suggest that increasing volume or viscosity will cause the same trends in WSS metrics, that CCs and SCs will cause broadly uniaxial and multidirectional flows, respectively, and that tilt will lead to symmetry breaking, over a wide range of baseline conditions. Consistent with this view, the values of OSI obtained with a CC by Driessen et al. (2020) never reached the values seen in the baseline configuration, without a cylinder, despite the wide range of conditions examined.

## 5 CONCLUSIONS

The study of endothelial responses to shear stress is important in the search for new ways of reducing or preventing atherosclerosis. The swirling well model has several practical advantages for such studies: chronic application of shear, the ability to produce different patterns of shear stress (including transverse flows), high throughput, low cost, and small fluid volumes. It also has disadvantages, of which the most important are the difficulty of separating effects of different shear metrics, and the fact that mediators secreted by cells exposed to one type of flow can alter the phenotype of cells elsewhere in the well, exposed to different flow regimes. The simulations conducted in the present study show that simple methods for modifying flow in the well, designed to maintain the advantages of the method, can overcome these issues. An example of the first issue is that TAWSS in the Control model is constant from the center of the well until a radial distance of 7 mm, where it starts rising, whereas transWSS remains constant from the center until 7 mm and then falls, making it impossible to distinguish effects of high TAWSS and low transWSS; here we showed that increasing the depth of medium abolishes the rise in TAWSS towards the edge. An example of solving the second type of issue is that purely multidirectional flow of constant magnitude was obtained everywhere under the SC, covering the large majority of the well, and its magnitude could be altered by changing the viscosity.

## AUTHOR CONTRIBUTIONS

**Mehwish Arshad**: Conceptualization; CFD studies; writing manuscript. **Shuyu Cheng**: CFD studies. **Maarten van Reeuwijk and Spencer J. Sherwin**: Conceptualization; advising on methods; reviewing manuscript. **Peter D. Weinberg**: Project leader; conceptualization; writing manuscript; obtaining funds.

## CONFLICT OF INTEREST

The authors declare no conflict of interest.

## Supporting information

Supporting information.

## Data Availability

Data are available from the corresponding author on reasonable request.
