# Supplementary material for: Modification of the swirling well cell culture model to alter shear stress metrics
Source: Biotechnol Bioeng. 2023 Jan 20;120(5):1254–68. doi: 10.1002/bit.28331 (PMC10952219; doi:10.1002/bit.28331)
Supplement: Supplementary file 1 — Supporting information. [file BIT-120-1254-s001.docx]

**Supplementary Material**

**
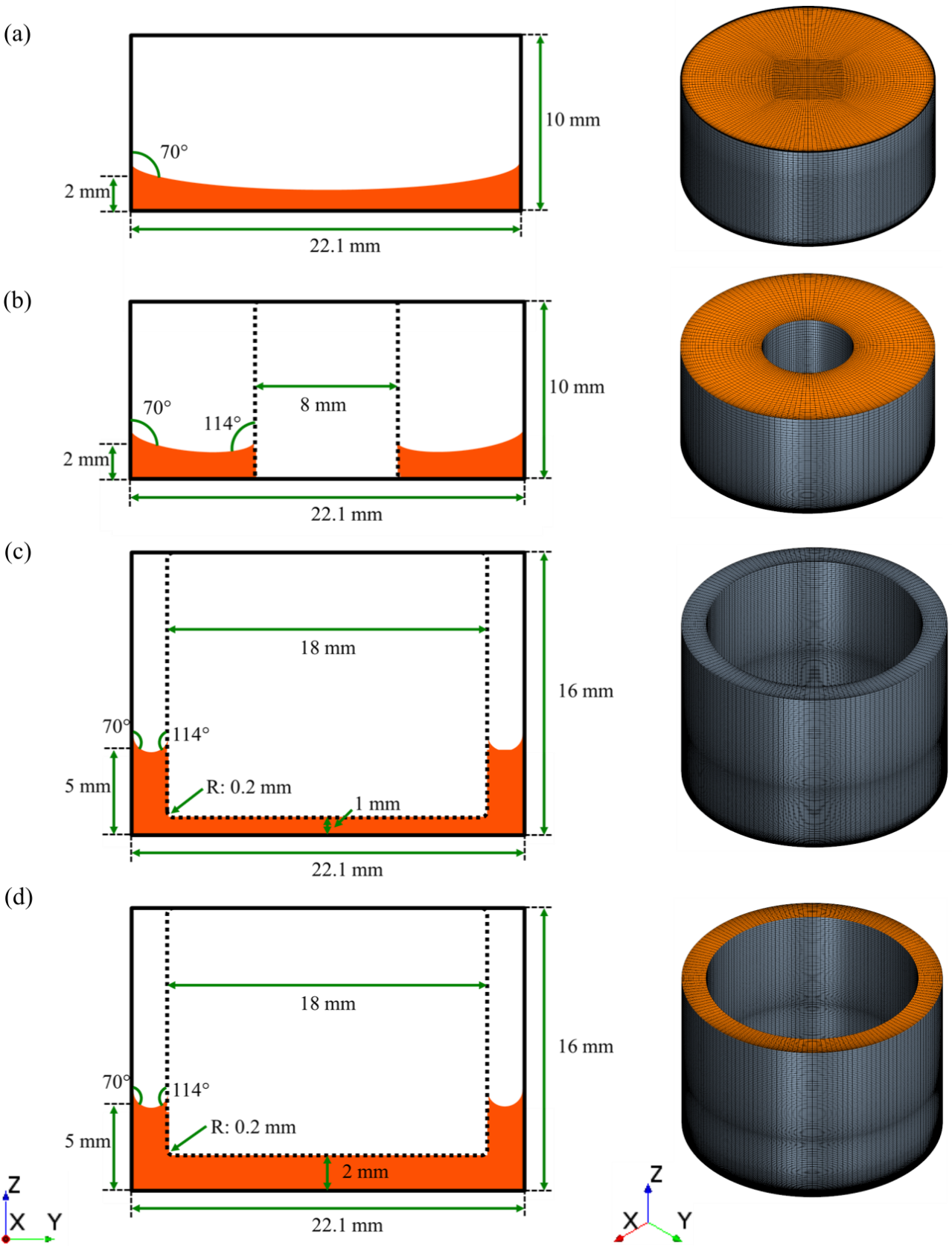
**

**Supplementary Figure 1** 2D geometry profiles and their respective mesh configuration for (a) Control model. The same geometry was used for the models with increased volume. (b) Central Cylinder model (c) Suspended Cylinder model with 1 mm gap. (d) Suspended Cylinder model with 2 mm gap. The same geometry was used for the models with increased viscosity.

**Steady state and transient study**

The maximum CFL numbers were <0.15 and <0.4, respectively, for all surface tension (**Supplementary Figure 2a**) and tilted models (**Supplementary Figure 3a**), thus satisfying the CFL<0.5 criterion. The mean liquid height remained within 0.5% starting value of all surface tension models (**Supplementary Figure 3b**).

Due to the periodic nature of flow within the well, the maximum and average WSS at the base of the well were expected to reach a periodic value after the initial transients dampened out and not to vary over time or show a repetitive pattern (**Supplementary Figure 2c-d** and **3b**). A time periodic state was reached after ~1.2 seconds (3 cycles of the shaker) for all models except volume = 1534 µl, which reached a steady state after ~4.0 seconds (10 cycles). Changes over time in the average WSS at the base of the well were negligible for all models once convergence was achieved (**Supplementary Figure 2d**). More obvious changes over time were seen in the maximum WSS (**Supplementary Figure 2c** 3b) which varied <6 mPa between the last two cycles for all models (**Supplementary Table 1**).


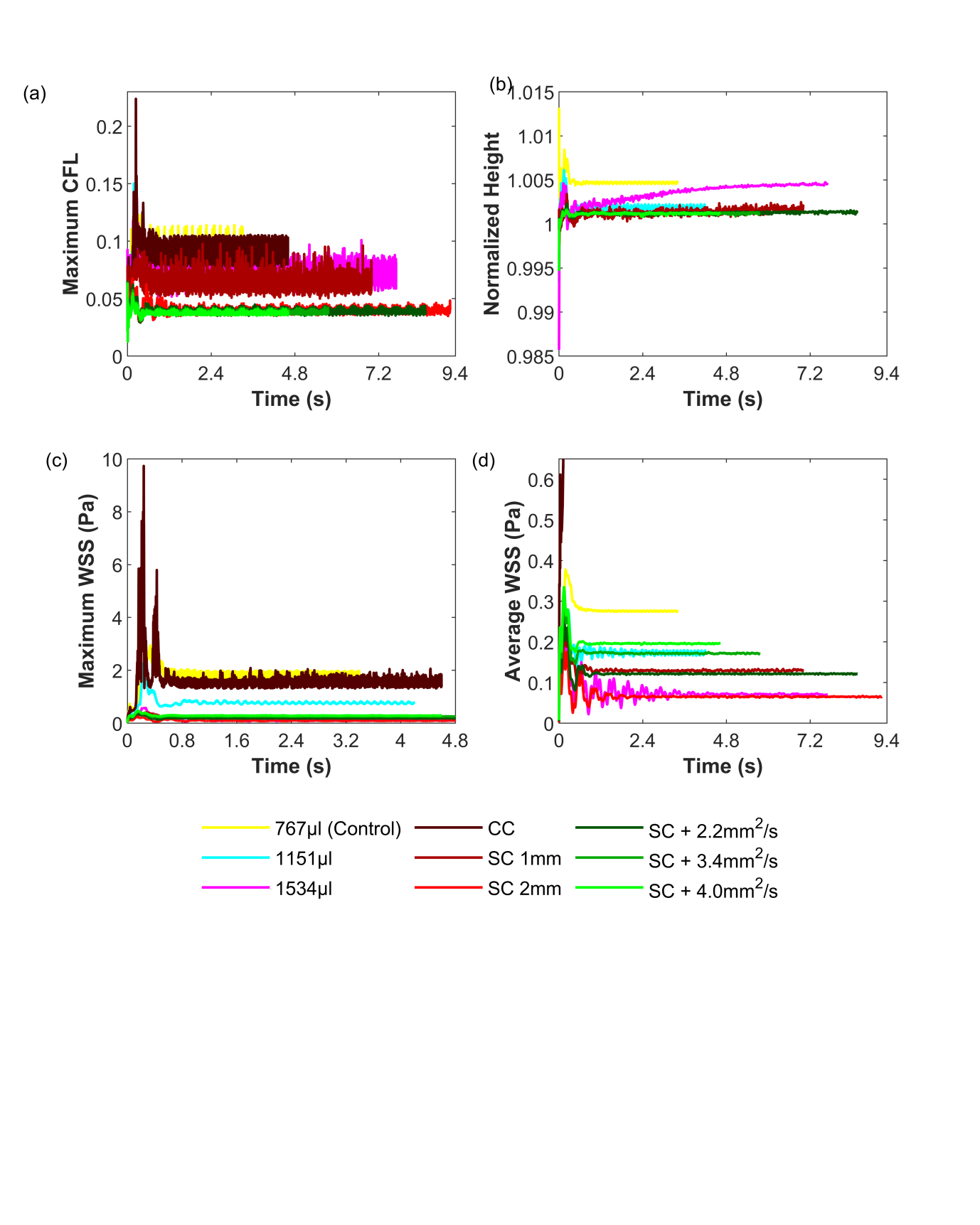

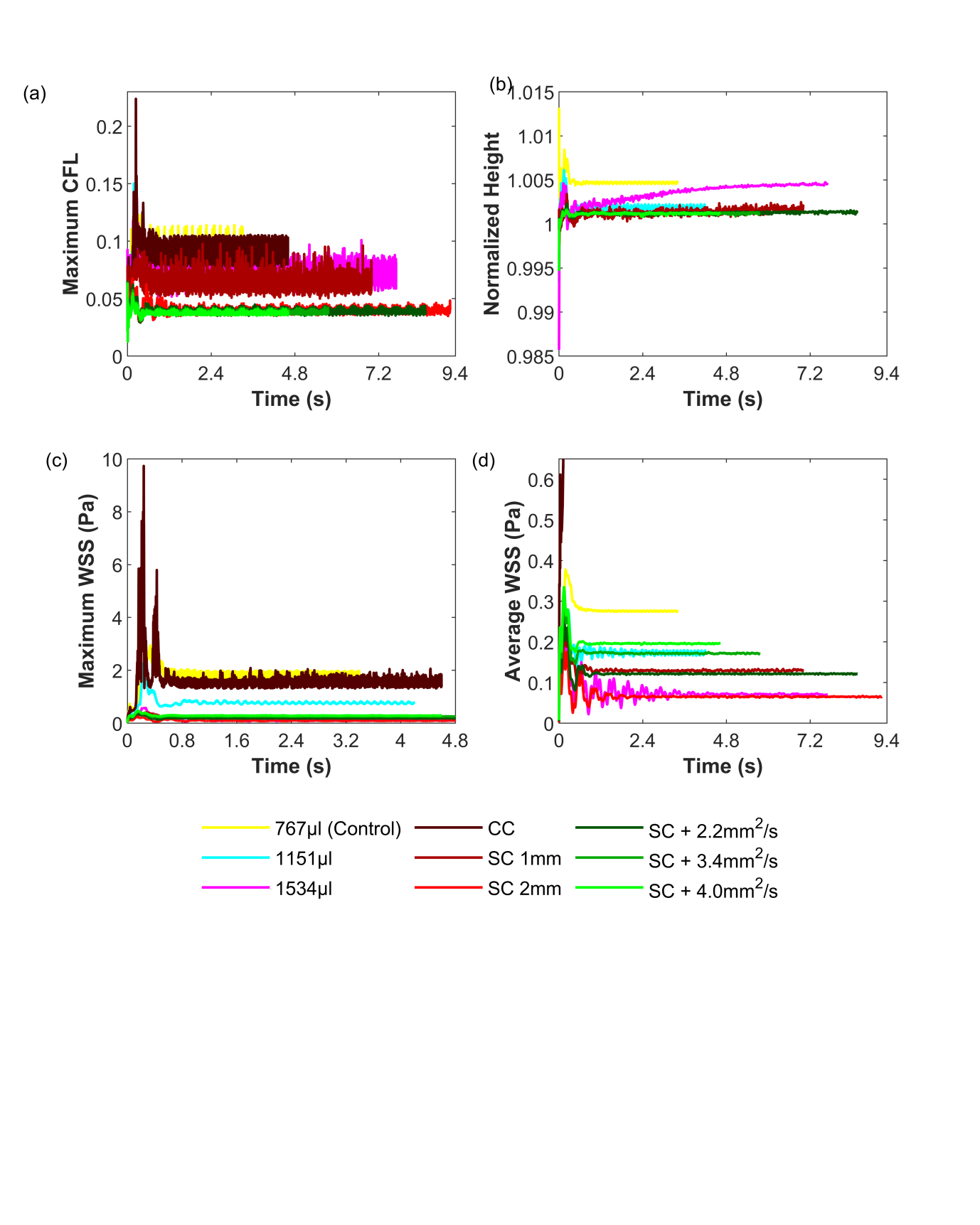


(a)

(b)

(c)

(d)

**Supplementary Figure 2** (a) Maximum CFL at the liquid-air interface for all modified surface tension models. The maximum CFL was below 0.2 for all models. (b) Average normalized height at the liquid-air interface (mean free surface elevation). The normalized values should remain at 1 to prevent any gain or loss of liquid volume during the simulations. (c) Maximum WSS at the base of the well as a function of time for all modified surface tension models. (d) Average WSS at the base of the well as a function of time for all modified surface tension models.


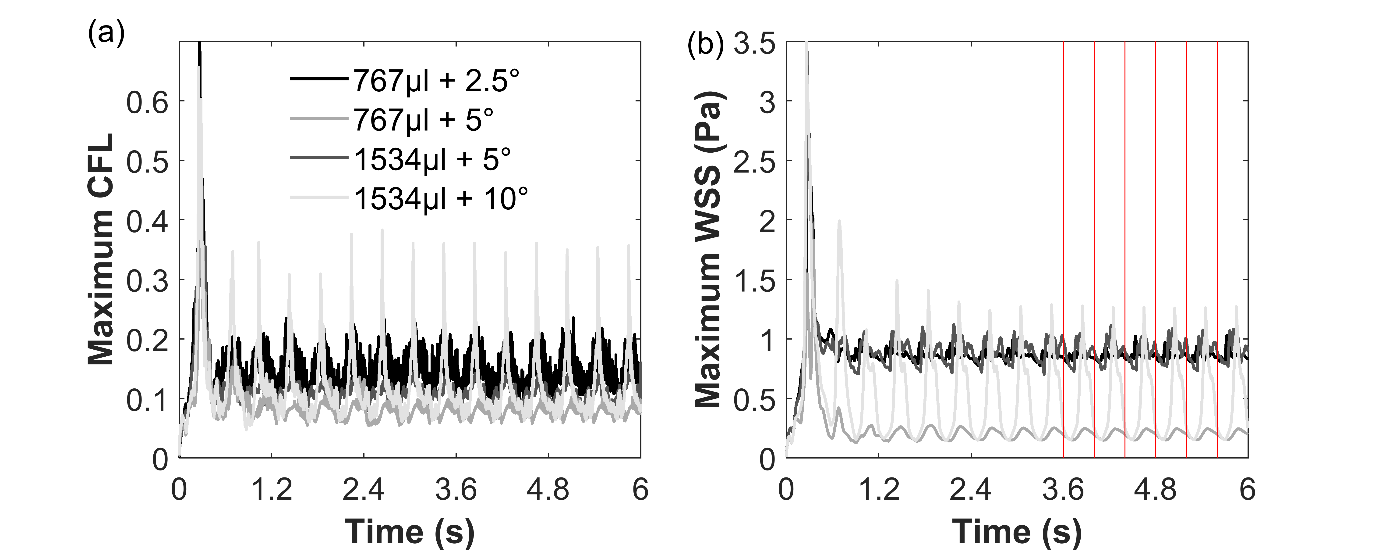


**Supplementary Figure 3** Temporal variation in (a) maximum CFL the at liquid-air interface for the tilted models, and (b) maximum WSS at the base of the well for the tilted models, which showed a repeating behaviour with a period of 0.4s (the red vertical lines are spaced 0.4s apart).

|  | **Minimum Steady State (s)** | **Total no. of cycles** | **Total time (s)** | **Max WSS variance (mPa)** |
| --- | --- | --- | --- | --- |
| Control | 0.8 | 7.0 | 3.4 | 1.0 |
| 1151 µl | 1.2 | 10.5 | 4.2 | 2.6 |
| 1534 µl | 4.0 | 13.0 | 6.2 | 2.3 |
| SC 1mm | 1.2 | 17.5 | 7.0 | 4.2 |
| SC 2mm | 2.4 | 23.0 | 9.2 | 0.1 |
| CC | 0.8 | 11.5 | 4.6 | 5.4 |
| SC+2.2mm^2^/s | 1.2 | 21.0 | 8.4 | 1.7 |
| SC+3.4mm^2^/s | 1.2 | 14.0 | 5.5 | 1.3 |
| SC+4.0mm^2^/s | 1.2 | 11.5 | 4.6 | 0.5 |

**Supplementary Table 1** Columns show, from L to R, the minimum time required to reach a steady state, the total number of cycles and total physical time for which the simulations were actually run (which was longer than required to reach a steady state), and the difference in maximum WSS between the final cycle, from which data were used, and the preceding cycle.


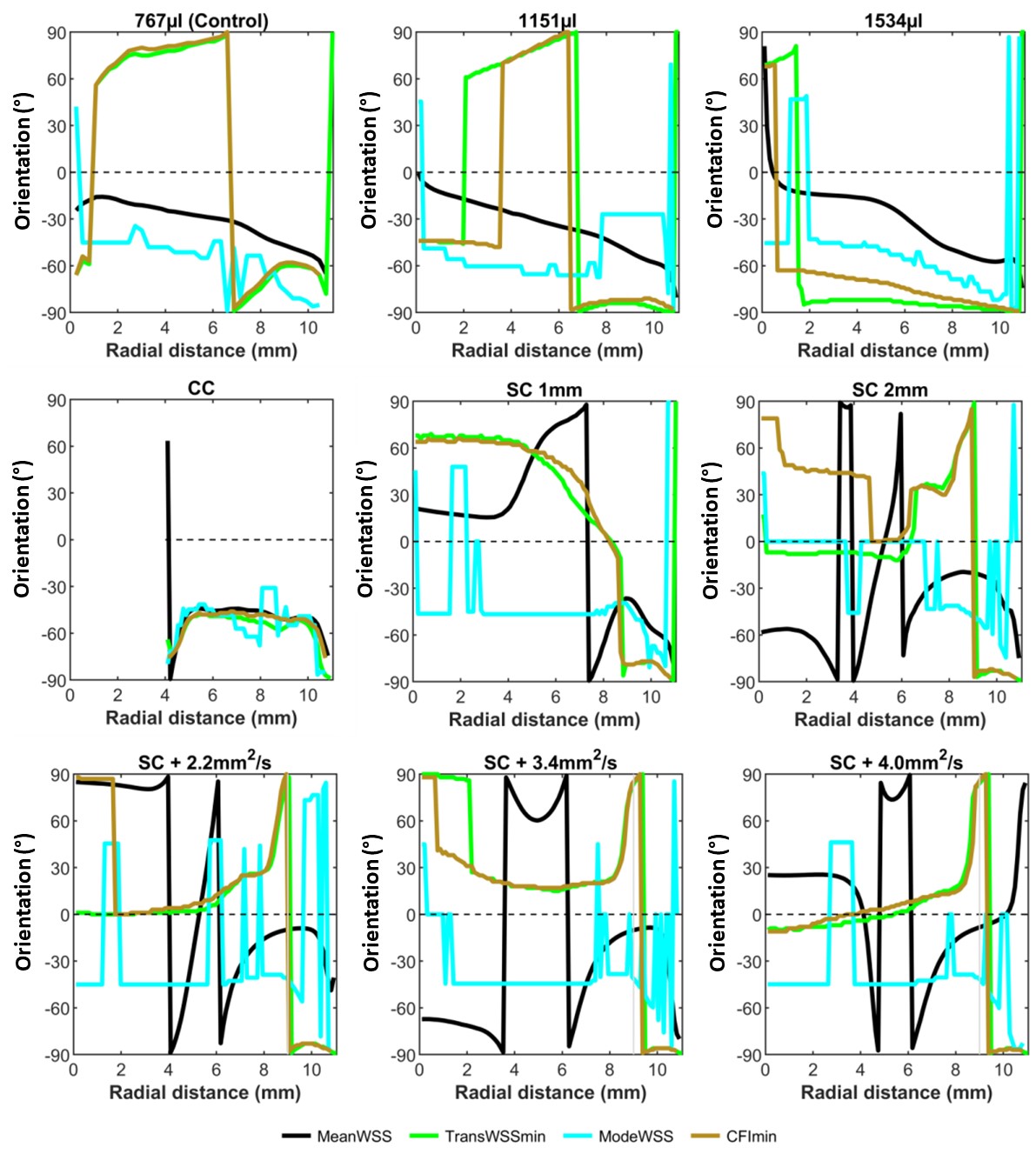


**Supplementary Figure 4** Directions of the mean and modal WSS vector and directions of the orientation, $\emptyset$, that minimises transWSS and CFI (defined in equations 11 and 12). Values are plotted from the centre to the edge of the well for the non-tilted models listed in the legend to Figure 2. Orientation was defined as the angle between the vector (or orientation) and the radial line at the same location, viewed from above. Angles clockwise from the radius were defined as positive. Angles were expressed from 0° to ±90° rather than 0° to ±180° (i.e. the vector was considered to be bidirectional along its axis, and the smaller angle was chosen) to allow comparison with studies of cell or nuclear orientation where the two ends of the cell or nucleus are not distinguished.
